# Salmagundi

**Published:** 1888-08

**Authors:** 


### SALMAGUNDI.

EDITED BY E. G. BETTY, D.D.S.

Death of Dr. A. Y. P. Garnet.—Dr. A. Y. P. Garnet, of Washington, ex-President of the American Medical Association, and for many years one of the most prominent members of the

Association, died at Atlantic City, N. Y., on Wednesday last. His admirable address before the Association at Cincinnati will be long remembered by those that heard it. Dr. Garnet was graduated from the university of Pennsylvania in 1841. His loss will be severely felt, not only in Washington City, but also by the whole profession.—*Journal Am. Med. Asso., July* 14.

---

THE Medico-Chiurgical College of Philadelphia and the Philadelphia Dental College have placed a contract for a large building to accommodate both schools.

---

OHIO STATE DENTAL SOCIETY REORGANIZED.—The next annual meeting of this society will be held in Cincinnati, October 16, 17, 18 inclusive. The local committee has succeeded in getting the magnificent assembly hall of the Lincoln Club in which to hold the sessions. The adjoining rooms will afford ample accommodations for the meeting of the Board of Directors, committees, etc. The Lincoln Club building is located at Garfield Place and Race St., just around the corner from the dental college, a locality all are more or less familiar with. The railroad fares will be down to the lowest notch, thus affording opportunity for dentists from all points to attend at the least expense. The Centennial Exposition of the Ohio Valley is an unrivaled attraction and will induce many to attend who could or would not do so otherwise. The entire profession is invited to attend and take part in the proceedings of the society. Valuable papers will be read by prominent members of the profession and the meeting will be of great profit to all.

---

EDUCATED CORPUSCLES.—"The future of preventive medicine," said Prof. Ray Lankester in the fascinating lecture which he delivered at the London institution, "is the education of the white blood corpuscle." A corpuscle is a minute cell of protoplasm which floats in the human blood. This minute creature eats and lives and flourishes and dies almost like a human being. Its special function, said the lecturer, is to eat up the poisonous element which finds its way into the blood. When a wound heals it is because these indefatigable corpuscles have found their way to the sore and have eaten away the injured part. When bacteria gets into the system the duty of the corpuscles is to go for them and eat them up. If they succeed, the patient recovers. If they are out of appetite, or the bacteria too tough a morsel for them to attack, the patient dies. Sometimes, with unconscious heroism worthy of Marcus Curtius, they purify the bodies in which they live by eating up poisonous particles and then ejecting themselves, thus sacrificing their own lives. But such heroic self-immolation is not necessary if you educate your corpuscle. His education proceeds by inoculation. By accustoming your protoplasmic cell to a low diet of mildly poisonous matter, such as the vaccine lymph, it becomes acclimatized, as it were, and is strong enough to eat up without inconvenience the germs of small-pox, which would otherwise prove fatal. It is these invaluable corpuscles which enable confirmed arsenic eaters to swallow with impunity a dose sufficient to kill six ordinary men, and Prof. Lankester is of the opinion that they can be trained so as to digest the most virulent poisons and deal with a great number of diseases.—*Pall Mall Gazette.*

